# Association of per- and polyfluoroalkyl substances (PFAS) exposure with cognitive function in firefighters

**DOI:** 10.1038/s41370-026-00861-y

**Published:** 2026-03-25

**Authors:** Xi Pan, Krystal J. Godri Pollitt, Sheng Liu, Felipe Armstrong, Bonny Amin

**Affiliations:** 1 Department of Sociology, Texas State University, San Marcos, TX USA; 2 Department of Environmental Health Sciences, Yale School of Public Health, New Haven, CT USA; 3 Department of Agricultural Sciences, Texas State University, San Marcos, TX USA

**Keywords:** PFAS exposure, Cognitive function, Firefighters, Workplace health

## Abstract

**Background:**

Evidence on cognitive function in relation to per- and polyfluoroalkyl substances (PFAS) exposure in firefighters is scarce.

**Objective:**

We conducted a pilot study aiming to explore the associations of individual PFAS analyte and PFAS mixture with domain-specific cognitive performance in firefighters.

**Methods:**

Firefighters (*n* = 65), who were actively serving four cities in Central Texas 2003–2025, were assessed for global cognition and processing speed measured by the NIH Toolbox Cognition Battery. Whole blood samples were collected from these participants and analyzed by liquid chromatography-tandem mass spectrometry for 24 PFAS analytes. Linear regression models were used to evaluate associations between cognitive performance and both individual PFAS analytes and the overall 24-PFAS mixture. Additionally, quantile g-computation and Bayesian Kernel Machine Regression (BKMR) were applied to investigate associations between 6-PFAS mixture and cognitive performance, with adjustment for potential covariates.

**Results:**

Six PFAS analytes including potassium perfluoro-1-octanesulfonate (PFOS), perfluoro-n-octanoic acid (PFOA), perfluoro-n-heptanoic acid (PFHpA), perfluoro-n-nonanoic acid (PFNA), perfluoro-n-decanoic acid (PFDA), and potassium perfluoro-1-hexanesulfonate (PFHxS) were detected in the least 70% of participants. PFOS was negatively associated with scores of processing speed (ß = –0.58 *p* < 0.001). Neither the 6-PFAS mixture nor the 24-PFAS mixture was associated with either cognitive domain.

**Significance:**

These preliminary findings may inform future research seeking to clarify the biological pathways linking PFAS exposure to cognitive outcomes and to explore possible modifiable factors related to cognitive health in firefighters. They might also underscore the potential value of continued effort to minimize workplace PFAS exposure for this occupational group.

**Impact:**

Our study provides the first data on PFAS exposure and cognitive performance for firefighters. Our analysis revealed that PFOS was the primary contributor among the PFAS mixture (consisting of PFOS, PFOA, PFHxS, PFHpA, PFNA, and PFDA) linked to reduced processing speed scores in firefighters. Our findings pave the way for future, larger studies that observe firefighters for longer duration to develop confident estimates of cognitive health in relation to PFAS exposure. These data could inform analyses in support of workplace exposure reduction efforts and revision of preventive health care for first responders.

## Introduction

Per- and polyfluoroalkyl substances (PFAS) are a class of synthetic fluorinated chemicals. Due to their unique physicochemical properties (e.g., non-stick, heat and acid resistant, waterproof, oil repellent, and stain resistant), PFAS have been widely used in consumer products and industry (e.g., surface coating and protectant formulations) since the 1950s [1]. Owing to their recalcitrant (non-biodegradable) and persistent (i.e., long half-lives) properties, PFAS are now called the “forever chemicals” and have potential for accumulation in human body [1, 2]. PFAS can enter the human body through inhalation, ingestion, and dermal absorption and accumulate in the body for years [3, 4]. Epidemiological studies have documented the adverse effect of legacy or the long-chain PFAS, such as perfluorooctane sulfonate (PFOS) and perfluorooctanoic acid (PFOA), on human health including cancer, immune suppression, thyroid dysfunction, metabolic effects, increased lipid levels, ulcerative colitis, liver and kidney function in adults [2, 5, 6] and neurodevelopment in children, including executive function deficits [7], cognitive dysfunction [8], and attention deficit and hyperactivity disorder [9]. Additionally, evidence from animal studies has suggested that PFOS and PFOA can reduce function of the nervous system and are potentially neurotoxic and correlate with neurobehavioral dysfunction, such as late-onset Alzheimer’s disease (AD) and learning and memory deficits [1, 10–12]. These studies have suggested two major potential pathways of PFAS neurotoxicity: (1) PFAS can disturb calcium homeostasis and induce calcium increase/overload in neurons. Calcium overload can disrupt the normal function of neurons and provoke neuronal excitement, which could lead to neuronal injury and cell death. In particular, PFAS tends to distribute to and accumulate in the hippocampus of the brain, a region implicated in learning and memory, and disturbs hippocampal neurons leading to cognitive impairment [13]; and (2) PFOS and PFOA can cross the blood-brain barrier (BBB) and alter neurotransmitter (e.g., dopamine, glutamate, and acetylcholine) concentrations which can influence the activation of neurons and signal transmission among neurons. Increases or decreases in neurotransmitters could lead to neuron cell death [1, 13].

Recent epidemiological studies have reported mixed findings on the relationship between cognitive function and serum PFAS in middle-aged and older adults in the United States [5, 14–16]. Shrestha et al. [14] found that higher serum PFOA and PFOS concentrations were associated with better performance in memory and learning, executive function, and visuospatial function among 126 adults aged 55–74 years old who lived in upper Hudson River communities. A cross-sectional study with 21,024 adults aged 50 and above, who lived in Mid-Ohio Valley, West Virginia, reported the protective impacts of PFOS, PFOA, and PFHxS on self-reported short-term memory [15]. A longitudinal study using data from participants from the National Health and Nutrition Examination Survey (NHANES; 2011–2014), a nationally representative sample of the U.S. population, suggested that a low level of exposure to a PFAS mixture (i.e., PFOA, PFOS, and PFHxS) is positively associated with cognitive performance in Americans aged 60 and above [5]. On the contrary, Park et al. [16], who studied the same NHANES population, revealed that higher levels of serum PFOS was associated with lower scores of global cognition after controlling for chronic kidney disease (CKD), non-monotonic dose-responses, and seafood consumption. Additionally, medical research has found that Alzheimer’s patients had significantly higher PFAS levels in their cerebrospinal fluid than non-AD patients [17], and high concentrations of PFOA have been found impairing dopaminergic neurons in the brain stems of autopsied adults, possibly impairing cognition [18]. While the body of literature on PFAS toxicity has expanded significantly in recent decades, it remains heavily skewed toward legacy PFAS (e.g., PFOS and PFOA) that have been phased out in many regions due to regulatory pressures. In contrast, studies examining the health impacts of PFAS mixtures—particularly those incorporating both legacy compounds and alternatives like shorter-chain analogs or fluorinated ethers [e.g., such as perfluorobutanoic acid (PFBA), perfluorohexanesulfonic acid (PFHxS), perfluorobutane sulfonic acid (PFBS), and perfluoroheptanoic acid (PFHpA)], often designed as less harmful substitutes (lower toxicity profiles and shorter environmental half-lives)—are notably underrepresented, highlighting a critical gap in understanding synergistic or cumulative effects in real-world exposure scenarios.

PFAS exposure may be of particular relevance to firefighting because these compounds are used in turnout gear, training activities in airports and military bases, and are a major ingredient of some firefighting foams, such as aqueous film-forming foams (AFFF) or Class B foam [19, 20]. AFFF are known to contain PFAS surfactants, including PFOS, PFOA, and PFHxS [2, 21]. Firefighters may be exposed to these PFAS compounds through inhalation of aerosolized foam, direct or indirect skin contact with contaminated personal protective equipment (PPE), as well as hand to mouth transfer during the use of AFFF [2, 22–27]. A number of biomonitoring studies have reported elevated serum PFAS concentrations in firefighters relative to the general population, likely related at least in part to occupational contact with PFAS-containing materials [20, 27–29]. Some studies have reported associations between PFAS exposure and increased risks of certain health issues—like cancer, kidney disease, and thyroid dysfunction—among firefighters, though the evidence is still being evaluated and causality has not been definitively established [30–32]. Additionally, firefighters might be under notable stress in their work due to the great workload and psychological stressors during fire and rescue operations. Unlike many other professions, firefighters are engaged in an occupation in which they are exposed to several stressors (e.g., night shift schedules, sleep deprivation, sudden alarm calls, strenuous physical work, exposure to smoke, heat, and other harmful substances during fire suppression) for an unforeseeable amount of time [33]. All the above factors can contribute to short-term and long-term impairment of mental health in firefighters [34]. Despite mounting concerns about firefighter health, few studies have assessed how exposure to PFAS may influence cognitive function in firefighters and cognitive health of firefighters is largely understudied worldwide. Such data are urgently needed given rapid population aging and the increase in the number of people having Alzheimer’s disease and related dementias (AD/ADRD). To address these notable gaps, objectives of this pilot study were to (1) identify and quantify concentrations of individual PFAS analyte and their mixture in blood, and (2) assess the association between PFAS and domain-specific cognitive performance in firefighters.

## Materials/subjects and methods

### Participants and recruitment

Participant recruitment, cognitive assessments, and general surveys capturing sociodemographic, basic health, and occupational characteristics were conducted between June 2023 and March 2025. Blood samples were collected between January and March 2025. Fire departments in Kyle, San Marcos, New Braunfels, and San Antonio in Texas assisted with the recruitment of firefighter participants by sending announcements through their listservs and newsletters. To recruit firefighter participants from San Antonio, a recruitment booth was established at the annual health fair in February 2024 and 2025 supported by the San Antonio Fire Department. Interested firefighters were invited to complete a screening survey to determine their eligibility for the study. Study inclusion criteria included career firefighters who were age of 18 and above, having at least 5 years of service at the city level, without a history of blood disease, central neurological disease, major psychiatric disorder, alcohol or substance abuse, serious medical illness (renal, hepatic, cardiac, or pulmonary insufficiency, cancer), or psychoactive drug use. In addition, career firefighters needed to be currently on “activity duty” at the time of recruitment. Following eligibility screening, 68 participants were scheduled for cognitive assessment and requested to complete a general survey. Of these, 65 were scheduled for blood collection, while 3 participants declined to provide their blood samples. Therefore, our final sample consisted of 65 participants with complete data. Participants who completed their cognitive assessment and provided their blood samples received a $60 gift card. All participants were consented into the study following protocols approved by the Institutional Review Board of Texas State University (#8639).

### Cognitive function assessment

Trained research staff conducted a 30–45-min cognitive assessment per standard protocols with each participant using an iPad in a reserved quiet room. We selected the NIH Toolbox Cognition Battery (NIHTB-CB) to assess the cognitive performance of our participants due to its validated sensitivity to domain-specific cognitive variations in a younger, healthier study population, including middle-aged individuals (ages 20–66 years), as demonstrated in recent validation studies [35, 36]. The Cognitive Battery is a computerized (iPad-based administration) neurobehavioral assessment consisting of multiple tests to measure cognitive domains including executive function, attention, episodic memory, language, processing speed, and working memory for a wide range of ages (7–85) [37, 38]. The Cognitive Battery has good scores on test-retest reliability, age effects on performance, and convergent and discriminant construct validity [37]. Following suggestions from neurologists and psychologists in our research team, we selected 7 tests to build our own battery to align with our research objectives and participant characteristics. The selected tests included Dimensional Change Card Sort (DCCS) Test for executive function, Flanker Inhibitory Control and Attention Test for attention, Picture Sequence Memory Test (PSMFA) for episodic memory, Pattern Comparison Processing Speed Test (PC) and Oral Symbol Digit (OSD) for processing speed, Face Name Associative Memory Exam (FNAMED) for associative memory, and List Sorting Working Memory Test (LSWM) for working memory. The new battery was piloted with our trained research staff to ensure clarity and functionality prior administration to study participants.

For each participant, an age-adjusted scale score for each individual test was produced by the Cognitive Battery after the administration. We calculated two summary scores to measure global cognition and processing speed. Calculation for the composite score of global cognition used all 7 tests, while computation for the composite score of processing speed used the PC and OSD tests. The composite scores were calculated by the average of the standardized z-scores of each test. Global cognition captures the overall cognitive function across multiple domains, reflecting the integrity of general cognitive abilities. Processing speed is a core component of cognitive function that allows first responders to handle information-rich, rapidly evolving situations, make critical decisions quickly, and perform their duties effectively under pressure [39]. Higher scores on these tests indicate better cognitive performance.

### PFAS exposure assessment

Capillary whole blood (via finger prick) as the sample matrix was selected to prioritize logistical feasibility. This method eliminated the requirement for on-site centrifugation, thereby minimizing the time burden on the firefighter participants and simplifying field collection. Moreover, several human studies have identified specific PFAS (e.g., FOSA, PFOSA, and PFHxA) that partition into red blood cells are found only in whole blood [40–42]. Blood samples were collected by trained research staff at the health fairs and fire stations where participants were assigned. The collection was done through finger prick using lancets (21 gauge and 2.2 depth) and vials. Each participant’s fingertip was disinfected with a 70% isopropanol alcohol wipe before sample collection. The first drop of blood was discarded to avoid PFAS contamination from disinfection products or finger prick needles. Three drops of blood (~150 µL) were collected into a heparin-containing 2 mL Eppendorf polypropylene vials (approximately 30 USP units per mL of whole blood). The vials were stored in a cooler with ice for transportation within 3 h from the fingerstick site (e.g., health fairs or fire stations) to Texas State University and stored at –20 °C in a biological lab until shipped on dry ice to the laboratory at the Yale School of Public Health for analysis. Once received by the laboratory, samples will be stored at -20 °C until analysis per standard protocols. The Data Use Agreement (DUA) on data sharing were signed by Texas State University and Yale University.

Whole blood (50 µL) was aliquot into a 2-mL Eppendorf microcentrifuge tubes and spiked with a mixture of mass-labeled PFAS internal standards (20 µL). Samples were vortexed and sonicated for 15 min. Methanol (130 µL) was added and then samples were vigorously shaken on a for 30 min. Samples were transferred to a polypropylene cellulose acetate filter tube and then centrifuged for 15 min to remove protein precipitation and cell debris. The supernatant was collected for each sample and transferred to a polypropylene autosampler vial for analysis of 24 PFAS analytes on an Agilent 1290 Infinity liquid chromatograph (LC) coupled to an Agilent 6545 quadrupole-time-of-flight mass spectrometer (LC-QTOF-MS) in negative electrospray ionization mode. Measured analytes included perfluorohexane sulfonic acid (PFHxS), perfluoroheptanoic acid (PFHpA), perfluorooctanoic acid (PFOA), perfluorooctane sulfonic acid (PFOS), perfluorononanoic acid (PFNA), perfluorodecanoic acid (PFDA), perfluorobutane sulfonic acid (PFBS), perfluoroundecanoic acid (PFUnDA), sodium perfluoro-1-heptanesulfonate (PFHpS), perfluorohexanoic acid (PFHxA), perfluoropentane sulfonic acid (PFPeS), perfluorooctane sulfonamide (PFOSA), perfluorododecanoic acid (PFDoA), perfluorobutanoic acid (PFBA), perfluoro-n-pentanoic acid (PFPeA), perfluoro-n-tridecanoic acid (PFTrDA), perfluoro-n-tetradecanoic acid (PFTeDA), sodium perfluoro-1-nonanesulfonate (PFNS), sodium perfluoro-1-decanesulfonate (PFDS), sodium 1H,1H,2H,2H-perfluoro-1-hexanesulfonate (4:2 FTS), sodium 1H,1H,2H,2H-perfluoro-1-octanesulfonate (6:2 FTS), sodium 1H,1H,2H,2H-perfluoro-1-decanesulfonate (8:2 FTS), N-methylperfluoro-1-octanesulfonamidoacetic acid [linear and branched] (MeFOSAA), and N-ethylperfluoro-1-octanesulfonamidoacetic acid [linear and branched] (EtFOSAA).

Samples were injected (10 µL) onto a InfinityLab Poroshell 120 EC C18 analytical column (2.1 mm × 100 mm × 2.7 µm; Agilent) preceded by a SecurityGuard C18 Guard Cartridge (4 mm × 2 mm I.D.; Phenomenex) and two Zorbax DIOL guard columns (4.6 mm × 12.5 mm × 6 µm; Agilent). LC binary pump flow rate was set at 0.4 mL/min and the starting mobile phase composition was 75% A (2.5 mM ammonium acetate in water) and 25% B (2.5 mM ammonium acetate in methanol) between 0 to 1 min. From 1 min to 3 min, the organic mobile phase B percentage increased from 25 to 75%. From 3 min to 8 min, the percentage of mobile phase B further increased from 75% to 100% and was held at 100% until 12 min, when the end of the run was reached. Data-dependent acquisition mode was used with iterative exclusion over mass-to-charge ratio (m/z) ratios between 118 and 1500 Dalton (Da) with fragmentation spectra collected at a fixed collision energy at 40 electron votes (eV) from 50 to 1500 Da. Dynamic exclusion was enabled so that a feature would be excluded after one spectrum collected and released after 0.5 min. The samples were analyzed along with a six-point calibration curve with native PFAS concentrations at 0.01, 0.05, 0.1, 0.5, 1, 2 parts per billion (ppb) limits of detection (LOD).

Of the 24 PFAS analytes, PFHxS, PFHpA, PFOA, PFOS, PFNA, and PFDA were selected for this study because the detection frequency (DF) of these PFAS were > 70% in our participants and they were also monitored in the U.S. general population in NHANES (Center for Disease Control, NHANES 2013–2014 Laboratory Data. https://www.n.cdc.gov/nchs/nhanes/search/datapage.aspx?Component=Laboratory&CycleBeginYear=2013). Levels of these PFAS analytes in this pilot study were measured in whole blood, whereas the NHANES reported concentrations in serum. Despite different biological matrices, levels of these PFAS analytes in this study could be compared to those measured in NHANES, but only after applying well-established conversion factors (e.g., a 2:1 serum/whole-blood ratio ± 0.2 depending on hematocrit and specific PFAS). PFDoDA, PFTrDA, PFTeDA, PFNS, PFDS, 4:2 FTS, 6:2FTS, MeFOSAA, and EtFOSAA were not detected, while 6 PFAS analytes including PFBA, PFPeA, PFBS, FOSA, 8:2 FTS, and PFHxA were measured in less than 20% of participants. We log-transformed concentrations of PFHxS, PFHpA, PFOA, PFOS, PFNA, and PFDA. To capture the combined exposure profile of 24 PFAS analytes, we calculated a composite score (a weighted sum of 24 PFAS analytes, Bayesian Weighted Sums [BWS)] for each participant representing the overall mixture exposure to PFAS, while accounting for uncertainty, non-detected values/left censoring, and correlations among the analytes. Given 9 undetected PFAS analytes, this calculation was performed using Bayesian hierarchical model with censoring through the PROC MCMC procedure in SAS. Bayesian hierarchical model with censoring is designed for treating undetected values as censored observations rather than missing or zero. The censoring mechanism in the hierarchical model directly handles this by modeling the latent (true) concentrations for censored values, ensuring their contributions (often small weights for undetected analytes) are included in the weighted sum. PFAS analytes exhibited a linear association with each cognitive outcome (see Figs. S3 and S4 in the Supplementary Material).

### Covariates

Sociodemographic and behavioral factors, basic health information, and occupational characteristics were obtained by self-reported questionnaires. A group of covariates was selected a priori based on experience in previous studies and a literature search: age (continuous, years), race (Non-Hispanic White vs. others), education (some college or less vs. Bachelor’s degree or above), annual individual income (≤$60,000 vs. >$60,000), smoking history (ever smoked cigarettes or electronic cigarettes), past alcohol consumption (average weekly standard drinks over the past year) and current consumption (average weekly standard drinks in the past 30 days), and depressive symptoms (Center for Epidemiologic Studies Depression Scale score ≥16 and/or use of antidepressant medication). We additionally considered seafood consumption (frequency of fish or shellfish consumption during the past 30 days: never, 1 to 3 times, ≥4 times) [16]. We also controlled for the potential impact of occupational characteristics including years of service at the city level, use of self-contained breathing apparatus (SCBA) last year, frequency of shower after a fire event, and frequency of use of firefighting foam for a fire event last year. We did not include gender as covariate due to the predominance of male firefighters in our sample.

### Statistical analysis

Power analysis was conducted to estimate statistical power with the sample of 65 using PROC POWER and MULTIREG statements in SAS. When the R-squared (R^2^) for the full regression model and the R^2^ difference for predictors of interest was set to 0.30 and 0.105, respectively; α error was set to 0.05 and the statistical power (1–β error) to 95%, the actual statistical power was moderate, 0.865.

Descriptive analyses were conducted to examine the sociodemographic characteristics of the participants, distributions of each PFAS analytes, and cognitive performance in the sample. Independent *t* tests were performed for continuous variables and Chi-squared tests for categorical variables. We calculated summary statistics including DF, arithmetic mean, standard deviation, and percentiles for each PFAS analytes.

To assess the associations of individual PFAS analyte (PFHxS, PFHpA, PFOA, PFOS, PFNA, and PFDA) and the 24-PFAS mixture with cognitive performance, we conducted separate sequential linear regression models to check if the relationship can be explained by different potential covariates. The initial model (Model 1) was the unadjusted model. Model 2 was based on Model 1 adjusting for age, race, education, income, cigarette smoking status, alcohol consumption, seafood consumption, and depressive symptoms. Model 3 controlled for variables in Model 2 as well as occupational characteristics: years of service, use of SCBA, frequency of shower after a fire event, and frequency of use of firefighting foam. We utilized PROC GENMOD procedure in SAS for model analyses.

We used quantile g-computation and Bayesian kernel machine regression (BKMR) to assess the association of the 6-PFAS mixture (PFHxS, PFHpA, PFOA, PFOS, PFNA, and PFDA) with cognitive performance. These methods can handle non-linearities, non-additive interactions among PFAS analytes, and variable selection in mixture, and estimate the joint association of a simultaneous one-quartile increase in the mixture with performance in each cognitive domain. We utilized *bkmr* package in R for model analyses.

In our sensitivity analyses, we applied generalized additive models (GAMs) with penalized splines to examine the potential non-linear associations between PFAS concentrations and cognitive domains. The PROC GAMPL procedure with Restricted Maximum Likelihood (REML) smoothing in SAS was used due to its more robust estimation for small samples.

## Results

As shown in Table 1, The mean age of our participants was 43 years (SD = 9.0), 92.3% were men, 56.9% were non-Hispanic White, and 49.2% with a Bachelor’s degree or above education. The average annual income was >$60,000. The average number of standard weekly alcohol or alcoholic beverage consumption was about 8 (SD = 1.2) over the past year and 1–7 (SD = 0.6) at present. Participants self-reported smoking cigarettes or electronic cigarettes (29.3%). In the past 30 days, 13.0% of participants reported not consuming any seafood, 32% indicated eating seafood 1 to 3 times, and 20% reported consuming it 4 or more times. For occupational characteristics, the average years of service was 11 years (SD = 6.8), 92.7% of participants reported having used SCBA last year, and 77.9% reported having one shower after a fire event. Additionally, 49.2% of participants reported that they occasionally used fluorine-free or Class A foam for a fire event last year, 33.8% indicated using most or frequently, and 7.7% reported using always. The average concentration of 24-PFAS mixture was 1.01 pg/μL (SD = 0.42). The composite z-score for global cognition had the mean of –0.002 (SD = 0.7) and ranged from –1.6 to 1.4, while the composite z-score for processing speed had the mean of 0.12 (SD = 0.78) and ranged from –1.86 to 2.45. Correlations between PFAS exposure and each covariate were demonstrated in Table S3 in Supplementary Material. Higher monthly seafood consumption was significantly associated with elevated PFOS concentration (*r* = 0.38, *p* < 0.01).

**Table 1:** Descriptive characteristics of firefighters in the study (*N* = 65).

	Mean ± SD	N(%)
Executive function (measured by DCCS)	104.1(13.2)	
Attention (measured by Flanker’s Inhibitory Control)	105.4(12.5)	
Associative memory (measured by FNAMED)	94.4(11.2)	
Working memory (measured by LSWM)	107.8(11.1)	
Episodic memory (measured by PSMFA)	111.8(10.8)	
Processing speed z score (measured by PC^†^ and OSD^‡^)	0.15(0.78)	
Global cognition z score (including all 7 tests)	0.01(0.69)	
Age (years)	42.8(9.0)	
Gender		
Men		60(92.3)
Women		5(7.7)
Race/ethnicity		
Non-Hispanic White		37(56.9)
Non-Hispanic Black		1(1.5)
Hispanic		26(40.0)
Multiracial		1(1.5)
Education		
Some college or less		33(50.8)
Bachelor’s or higher		32(49.2)
Annual individual income		
≤$60,000		8(12.3)
>$60,000		57(87.7)
Ever Smoked cigarettes or e-cigarettes		19(29.2)
Number of weekly alcohol consumption		
Past	2.7(1.2)	
Currently	1.9(0.6)	
Frequency of seafood consumption in the past 30 days		
Never		13(20.0)
1–3 times		32(49.2)
4 times or more		20(30.8)
Having depressive symptoms*		
Yes (CES-D scores ≥16 or use of antidepressant medication)		53(81.5)
No (CES-D scores <16)		12(18.5)
Years of service at the city level	11.3(6.8)	
Reported using firefighting foam in the past year		
Never		6(9.2)
Sometimes/occasionally		32(49.2)
Most of the time/frequently		22(33.8)
Always		5(7.7)
Shower frequency after a fire event		
Once		50(76.9)
Twice or more		15(23.1)
Used self-contained breathing apparatus (SCBA) during salvage/overhaul		
Yes		60(92.3)
No		5(7.7)

*DCCS* Dimensional Change Card Sort Test, *PSMFA* Picture Sequence Memory Test, *FNAMED* Face Name Associative Memory Exam, *LSWM* List Sorting Working Memory Test.

* CES-D: Center for Epidemiologic Studies Depression Scale, 10th Edition. ^†^PC: Pattern Comparison Processing Seepd. Age-adjusted score range of PC: 0–150. ^‡^OSD: Oral Symbol Digit. Age-adjusted score range of OSD: 0–144.

Table 2 shows that among the 24 PFAS analytes, PFHpA, PFOA, PFOS, and PFHxS were detected in 100% of study participants, while PFNA and PFDA were detected in 93.75% and 77.78%, respectively. The arithmetic means of blood concentrations were 0.40 pg/μL (SD = 0.31) for PFHpA; 0.56 pg/μL (SD = 0.27) for PFOA; 2.4 pg/μL (SD = 1.24) for PFOS; 0.90 pg/μL (SD = 0.57) for PFHxS; 0.16 pg/μL (SD = 1.00) for PFNA; and 0.07 pg/μL (SD = 0.05) for PFDA. Distributional comparisons of these 6 PFAS analytes between this study and the NHANES were demonstrated in Figure S1 (Supplementary Materials). Details of the distributions of 24 PFAS in this study and their comparison with NHANES are presented in Table S1. Table S2 showed that 5(7.7%) firefighters who reported always using firefighting foam in the past year had higher levels of all 6 PFAS analytes than those who reported not using firefighting foams in the past year. In particular, PFHxS and PFOS levels were 2 times higher in these firefighters compared to firefighters who did not use firefighting foam in the past year before our study.

**Table 2:** Descriptive characteristics of PFAS concentrations (pg/μL) analyzed in the study.

		DF %	LOD	Mean	SD	25th	50th	75th
Potassium perfluoro-1-octanesulfonate (Linear and Branched)	PFOS	100	0.06	2.40	1.24	1.53	2.13	2.95
Perfluoro-n-octanoic acid (Linear and Branched)	PFOA	100	0.04	0.56	0.27	0.36	0.49	0.78
Perfluoro-n-heptanoic acid	PFHpA	100	0.49	0.40	0.31	0.19	0.31	0.50
Perfluoro-n-nonanoic acid	PFNA	93.75	0.03	0.16	1.00	0.09	0.15	0.22
Perfluoro-n-decanoic acid	PFDA	77.78	0.08	0.07	0.05	0.03	0.08	0.10
Potassium perfluoro-1-hexanesulfonate	PFHxS	100	0.01	0.90	0.57	0.49	0.76	1.19
24-PFAS mixture^*^				1.01	0.42	0.75	0.89	1.26

^*^Composite scores for a mixture of 24 PFAS (per- and polyfluoroalkyl substances) analytes using Bayesian Weighted Sums (BWS) through the PROC MCMC procedure in SAS.

Results from linear regression models assessing the relationship between PFAS concentrations and cognitive performance in both unadjusted and adjusted models are presented in Table 3. For PFOS, the absolute value of the correlation coefficient increased progressively from the unadjusted Model 1 to the fully adjusted Model 3 (β =–0.13 to –0.58 for processing speed; β = –0.13 to –0.36 for global cognition), indicating a stronger and more statistically significant association between exposure to these PFAS analytes and cognitive performance after adjustment for covariates. After adjusting for the potential covariates, PFOS was negatively associated with processing speed (ß = –0.58, *p* < 0.001). For a 10-fold increase in levels of PFOS (e.g., 0.1 pg/μL to 1 pg/μL), there was 0.58 SD (8.7 points) decrease in processing speed, with the test having a SD of 15 points. PFOS was not associated with global cognition. PFOA, PFNA, PFDA, PFHpA, or PFHxS was not individually associated with either cognitive domain. Additionally, 24-PFAS mixture was not associated with global cognition or processing speed.

**Table 3:** Effect estimates (β) and 95% Confidence Intervals (CIs) of Global Cognition and Processing Speed per tenFold Increase in PFAS Concentrations in Whole Blood among 65 Firefighter Participants, Multiple Linear Regression Models.

	Global cognition		Processing speed	
	β (95% CI)	*P* value	β (95% CI)	*P* value
PFOS				
Model 1	–0.13(–0.42 to 0.16)	0.38	–0.13(–0.42 to 0.16)	0.22
Model 2	–0.27(–0.62 to 0.08)	0.13	**–0.38(–0.76 to –0.003)**	**0.048**
Model 3	–0.36(–0.72 to –0.01)	0.05	**–0.58(–0.91 to –0.24)**	**<0.001**
PFOA				
Model 1	0.03(–0.34 to 0.40)	0.89	0.20(–0.19 to 0.60)	0.32
Model 2	0.16(–0.25 to 0.57)	0.45	0.21(–0.23 to 0.65)	0.35
Model 3	0.12(–0.30 to 0.54)	0.58	0.06(–0.36 to 0.47)	0.78
PFHpA				
Model 1	–0.15(–0.42 to 0.12)	0.27	–0.12(–0.41 to 0.18)	0.44
Model 2	–0.20(–0.49 to 0.08)	0.17	–0.15(–0.46 to 0.16)	0.35
Model 3	–0.22(–0.50 to 0.07)	0.14	–0.16(–0.45 to 0.12)	0.27
PFNA				
Model 1	–0.04(–0.28 to 0.19)	0.72	0.11(–0.14 to 0.37)	0.37
Model 2	–0.06(–0.31 to 0.19)	0.63	0.19(–0.08 to 0.46)	0.16
Model 3	–0.13(–0.40 to 0.13)	0.33	0.06(–0.20 to 0.33)	0.63
PFDA				
Model 1	–0.04(–0.18 to 0.09)	0.52	–0.10(–0.24 to 0.05)	0.19
Model 2	–0.01(–0.15 to 0.13)	0.88	–0.10(–0.25 to 0.05)	0.18
Model 3	–0.004(–0.15 to 0.14)	0.96	–0.08(–0.22 to 0.06)	0.28
PFHxS				
Model 1	–0.12(–0.40 to 0.16)	0.40	0.10(–0.21 to 0.40)	0.53
Model 2	–0.16(–0.47 to 0.15)	0.32	0.06(–0.28 to 0.40)	0.72
Model 3	–0.20(–0.53 to 0.12)	0.22	–0.05(–0.38 to 0.27)	0.75
24-PFAS mixture				
Model 1	0.34(–0.06 to 0.74)	0.10	0.72(0.30–1.15)	0.10
Model 2	0.16(–0.28 to 0.60)	0.49	**0.55(0.09–1.01)**	**0.02**
Model 3	0.16(–0.31 to –0.64)	0.50	0.42(–0.04 to 0.87)	0.08

a. Model 1: unadjusted model. b. Model 2: Model 1+ age, race/ethnicity, education, annual individual income, smoking history, number of past and current alcohol consumption, frequency of seafood consumption, and depressive symptoms. c. Model 3: Model 2+occupational characteristics: years of service, frequency of shower, frequency of firefighting foam utilization, and use of Self-Contained Breathing Apparatus (SCBA).

*PFOA* perfluorooctanoic acid, *PFNA* perfluorononanoic acid, *PFDA* perfluorodecanoic acid. *PFHpA* perfluoroheptanoic acid, *PFHxS* perfluorohexanesulfonic acid (or perfluorohexane sulfonic acid).

**Fig. 1:**
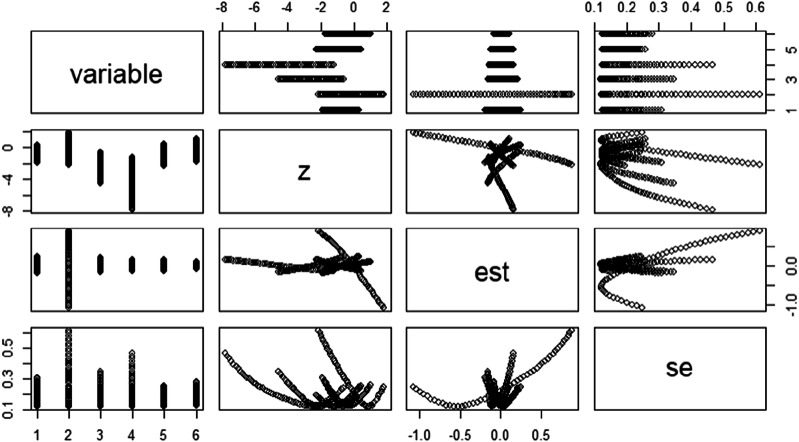
Plots based on Bayesian Kernel machine regression (BKMR) to exhibit the association between 6-PFAS mixture and processing speed. “est” column is the posterior mean estimate to quantify how the exposure to PFAS mixture influences processing speed.

**Fig. 2:**
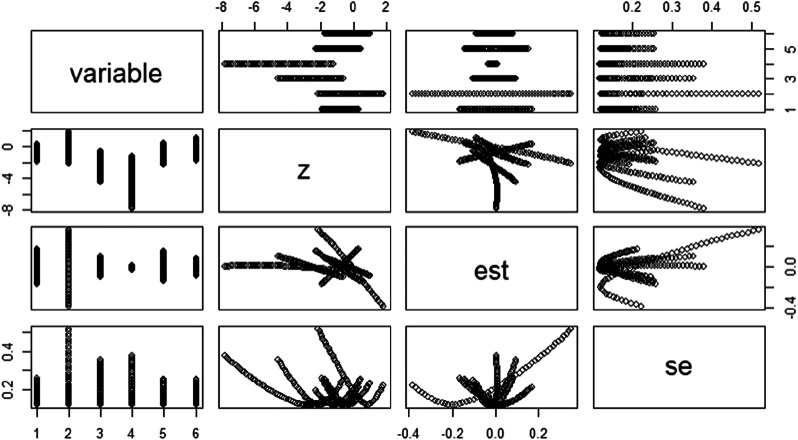
Plots based on Bayesian Kernel Machine Regression (BKMR) to exhibit the association between 6-PFAS mixture and global cognition. “est” column is the posterior mean estimate to quantify how the exposure to PFAS mixture influences global cognition.

Results from the quantile g-computation and BKMR investigating the associations of 6-PFAS mixture and each cognitive domain were presented in Table 4. The Markov Chain Monte Carlo (MCMC) diagnostics indicated satisfactory acceptance rates for the kernel parameters in the model examining associations between the 6-PFAS mixture and cognitive domains (r = 0.34 for processing speed; 0.37 for global cognition). The chains exhibited good mixing (λ = 0.34) for processing speed, indicating efficient sampling and supporting reliable posterior inferences. In contrast, mixing was suboptimal for global cognition (λ = 0.21), suggesting less efficient exploration of the posterior and weaker support for reliable inference. Moreover, the overall mixture exhibited no significant association with processing speed as the credible interval for the primary comparison (75th vs. 25th) included 0. Posterior mean estimates were small and displayed opposing directions in opposite directions (slightly positive at lower exposure levels and slightly negative at higher levels), while the associated uncertainty (posterior standard deviations, SDs) exceeded the magnitude of the estimates themselves. However, this null overall mixture effect appeared to be driven primarily by heterogeneity across individual PFAS analytes: PFOS exhibited a strong association with processing speed (posterior inclusion probability [PIP] = 0.80), whereas the PIPs for the other PFAS analytes were substantially weaker (PIPs ≈ 0.3–0.5), suggesting that the estimated joint effect of the mixture was dominated by PFOS. These findings were further corroborated by visualizations of the exposure-response function *h*(z), which depict the estimated relationship between the overall PFAS mixture and cognitive outcomes (see Figs. 1 and 2).

**Table 4:** Bayesian Kernel machine regression (BKMR) and quantile G-computation for assessing the associations of 6-PFAS mixture with cognitive domain performance.

	Processing speed			Global cognition		
BKMR	PIPs	λ^*^	r (overall acceptance rate)	PIPs	λ	r (overall acceptance rate)
		0.34	0.34		0.21	0.37
PFOS	0.80				0.46	
PFOA	0.44				0.39	
PFNA	0.33				0.35	
PFDA	0.20				0.23	
PFHpA	0.33				0.41	
PFHxS	0.32				0.37	
**Quantile g-Computation**						
		**Posterior mean estimates**	**SD**		**Posterior mean estimates**	**SD**
95% CI^†^	–0.65 to –0.12			–0.57 to 0.11		
Quantile	25th	0.13	0.14		0.12	0.12
	75th	–0.14	0.13		–0.11	0.12

^*^ λ: the variance component (or signal variance) in the Gaussian kernel that defines the exposure-outcome function *h*(z), estimated via the Markov Chain Monte Carlo (MCMC) sampling process. ^†^95% CI: 95% confidence interval from the posterior distribution to quantify uncertainty and assess (e.g., the change in outcome when exposure shifts from the 25th to the 75th percentile) the significance of mixture exposure-outcome association. Significant 95% CI should not include 0.

*BKMR* Bayesian Kernel Machine Regression, *PIPs* posterior inclusion probabilities, *PFOS* perfluorooctanesulfonic acid (or perfluorooctane sulfonic acid), *PFOA* perfluorooctanoic acid, *PFNA *perfluorononanoic acid, *PFDA* perfluorodecanoic acid, *PFHpA* perfluoroheptanoic acid, *PFHxS* perfluorohexanesulfonic acid (or perfluorohexane sulfonic acid), *SD* posterior standard deviation.

In sensitivity analyses, we evaluated potential non-linearity using smoothing plots shown in Fig. S2 (see the Supplementary Materials). The smoothing component panel visualized the spline transformations for PFOS, PFOA, PFNA, PFDA, PFHxS, PFHpA, and 24-PFAS mixture in addition to 95% Bayesian curvewise confidence bands. There were clear linear negative associations of PFOS and 24-PFAS mixture with z-scored global cognition and processing speed. The results were consistent with those of linear regression models.

## Discussion

This pilot study is one of the first epidemiological studies to assess the association between PFAS exposure and cognitive performance in firefighters. Of the 24 PFAS we measured, detection frequencies ranged from 0 to 100% and all participants had at least 6 PFAS analytes (PFHxS, PFHpA, PFOA, PFOS, PFNA, and PFDA) detected in their whole blood samples. Our findings suggest that levels of PFOS had a moderate-to-large effect, potentially impacting occupational performance in tasks requiring rapid processing abilities, which are critical for firefighting. The association is statistically significant, but the low overall PFAS levels (<1 pg/μL) suggest high sensitivity to small exposure changes. For individual firefighters, this decline may affect job efficacy but is unlikely to indicate clinical impairment unless cumulative. For the firefighter population, it supports exposure reduction and cognitive monitoring. Additionally, the magnitude of the correlation coefficients increased progressively from the unadjusted Model 1 to the fully adjusted Model 3 (which included occupational characteristics). This strengthening of associations after covariate adjustment highlights the potential for confounding bias in studies of PFAS exposure and cognitive outcomes when key occupational factors are not considered.

These findings align with evidence suggested by prior epidemiological studies, [15–17]which report that neurotoxicity of PFAS varies by carbon chain lengths and the functional group: larger adverse effects were exhibited in legacy or long-chain PFAS (e.g., PFOS and PFOA, compared to alternative shorter-chain PFAS) and the sulfonate functional group [16, 43]. In cell-cultured rat models, Berntsen et al. (2017) [44] also confirmed that the cytotoxicity of neurons increases with increasing carbon chain lengths and the sulfonate functional group and leads to a greater toxicity than the carboxylate group if the chain length is the same. Additionally, PFOS exhibits a lower elimination rate from the body compared to other PFAS analytes due to its longer half-lives or biological persistence [17, 45]. In our study, the average concentration of PFOS was the highest among the 6 PFAS analytes (x̄=2.40 pg/μL), and its concentrations at each quartile (25th, 50th, and 75th percentiles) were also consistently the highest (1.53 pg/μL for the 25th, 2.13 pg/μL for the 50th, and 2.95 pg/μL for the 75th) as shown in Table 2. Even after the 2002 voluntary phase-out of PFOS production, legacy foams remain in use or contaminate sites, equipment, and turnout gear. PFOA was present in much lower amounts (often <10%) in these foams, either as an impurity or byproduct [2, 45]. Additional sources like PFAS-treated turnout gear may contribute more to PFOS migration through layers during use or laundering. These could be the potential mechanism to explain the adverse impact of PFOS on processing speed over PFOA in our study as well as the NHANES study for the general population [16].

Our findings add to the current literature in providing evidence that PFOS exerted the predominant influence within the PFAS mixture on processing speed among firefighters. These results are consistent with the findings of similar prior research studied PFAS concentrations on neurohealth in early and late life [17, 46]. In an exploratory study of older French cohort at a hospital setting, branched PFOS was found significantly higher among 8 older patients with both clinical and biological signs of neurological impairment than those without [17]. A literature review on the impact of PFAS exposure on neurodevelopment in early life suggests that the legacy PFAS concentrations, such as PFOS, PFHxS, and PFDA, are persistently associated with lower trajectories of cognitive function including grow motor and problem solving in children between 3 and 36 months [46]. The potential mechanism for these results is that legacy PFAS analytes can freely cross the BBB and have significant accumulation in brain, which disrupts calcium homeostasis and dopamine and glutamate signaling, and consequently affects cognitive functions and contributes to unfavorable cognitive performance [46].

We did not find significant associations of cognitive domains with either the 6-PFAS mixture or the 24-PFAS mixture in this firefighter population, after adjusting for the occupational characteristics. This finding is not unexpected, as it can be attributed to two primary factors. First, epidemiological evidence on childhood PFAS exposure and neurodevelopmental scores suggests that despite the adverse cognitive outcomes in relation to PFAS mixture, legacy PFAS were major contributors that drove the mixture associations, with little contribution from alternative PFAS [46, 47]. Second, the detection rates of alternative PFAS were low in this population resulting in their minimal impact on cognitive domains.

Widespread use for PFAS in consumer products, contamination of food and water sources, and their environmental persistence may contribute to the high levels of exposure in firefighters. Turnout gear may be a major source of PFAS exposure in firefighters in our study. Recent studies have shown that PFAS are present in all three layers of firefighter turnout gear: the outer shell, moisture barrier, and thermal liner [25, 48]. These studies highlight the health risks associated with the materials and finishes used in turnout gear even before it is exposed to its first fire. In our study, firefighters all wear turnout gear during their firefighting, overhaul, and working in smoke. Although clean procedures, including basic spot cleaning, full wash, and specialized for heavy chemicals, that generally have been performed to reduce the PFAS exposure before and after fire or rescue events, PFAS residues may persist on turnout gear and can penetrate the skin through dermal absorption, entering the bloodstream and potentially impacting cognitive function. In particular, insufficient processing speed could slow their responses, increase the risk of errors, and jeopardize both rescue missions and personal safety. Firefighting foams may be another important source of PFAS exposure. In our study, 61 firefighters reported that they have only used fluorine-free or Class A foam after their training in the past year before our study, while 4 firefighters reported that they used both Class A and AFFF. A study in Finland found that firefighters using AFFF in training activities had increased levels of PFHxS in their blood after the training activities [19], however we lacked access to valid, individual-specific data on the types or chemical compositions of firefighting foams to which participants were exposed. As a result, non-differential exposure misclassification likely attenuated the observed associations meaning our reported effect estimates were probably conservative.

Following adjustment using a 2:1 serum/whole-blood conversion factor, comparison of seven PFAS analytes (PFOA, PFNA, PFDA, PFUnDA, PFHxS, PFHpS, and PFOS) revealed that firefighters in the present study had elevated PFHxS and PFOS concentrations compared with the NHANES 2013–2014 cohort. PFDA and PFUnDA concentrations were nearly identical to those reported in NHANES, whereas PFOA, PFNA, and PFHpS levels were slightly lower. The NHANES data were collected 2013–2014 and our samples were collected 2023-2025. This may reflect the temporal trends associated with the phase-out of these compounds in consumer products and firefighting equipment [2]. Additionally, firefighter participants in our study were without CKD, while HNANES participants included those with CKD. CKD may result in higher serum concentration for specific PFAS analytes [16, 49].

Our study has several limitations. First, the absence of a significant association between PFAS exposure and global cognition may be attributable, at least in part, to the healthy worker effect. Because our cohort consisted of active-duty career firefighters, a population subject to rigorous pre-employment screening and ongoing medical surveillance, individuals with poorer baseline cognitive function or neurological health are less likely to enter or remain in this occupation, potentially rendering them less susceptible to or less likely to exhibit PFAS-global cognition deficits within the observed exposure range. Second, the overall small sample size (N = 65) may diminish statistical power to assess the relationship between the 24-PFAS mixture and cognitive performance. Given the complexity of mixtures, larger sample sizes are needed to produce reliable estimates. Third, we could not investigate differences in the PFAS-cognition relationship by specific racial/ethnic group and disparities among them. Gender heterogeneity in the PFAS-cognition relationship was also not able to be examined due to the predominance of male firefighters in our sample. Generalizability of our findings should be tested with a large and diverse sample. Fourth, cross-sectional study design cannot draw causal inference for the impact of PFAS exposure on cognitive performance. Epidemiological studies of longitudinal trajectory of PFAS exposure and cognitive function are urgently needed to replicate our findings. Moreover, control groups, such as office workers who were not first responders, need to be included to differentiate occupational risks in relation to PFAS exposure. Fifth, we adjusted the data for most identified and measured potential confounders based on the limited literature, but we cannot rule out biases due to unmeasured confounders. For example, it was difficult to assess the association between PFAS exposure and fire events. Although our firefighters had responded to a fire event in the 24 h prior to the sample collection. We also did not have information on the intensity of the fire, the amount of firefighting foam used at a fire event, the formulations of firefighting foam, or the participants assigned role at the fire event (e.g., whether the firefighter was involved in direct fire suppression activities or providing back up and support). Additionally, all occupational characteristics for firefighters were based on self-reported data, which is often influenced by recall bias.

Despite these limitations, our study is the first epidemiological study to explore the association between PFAS exposure and cognitive performance in firefighters. This study fills the data gaps and contributes to the existing environmental health literature in enriching and replenishing the examination of environment–cognition relationship in firefighters and providing the basis for future research to elucidate mechanisms of PFAS affecting domain-specific cognitive function and subsequently characterize potential modifiable risks for cognitive impairment and AD/ADRD. Moreover, findings of the study highlight opportunities to develop effective intervention and prevention strategies to reduce workplace exposure for firefighters. These findings will also educate health care professionals about the relationship between workplace exposure and cognitive function, and promote neurocognitive screening tests and assessment as a part of routine exams for firefighters to reduce their risk for developing dementia. Finally, our findings inform the risk assessment process used by government officials to derive health-based guidelines and standards that regulate PFAS in environment and protect the public from potential unsafe level of exposure.

In conclusion, our study provides the first data on PFAS exposure and cognitive performance for firefighters. We found that PFOS was the primary contributor among the PFAS mixture (consisting of PFOS, PFOA, PFHxS, PFHpA, PFNA, and PFDA) linked to reduced processing speed scores in firefighters. While confirmative studies are required, our findings recommend interventions to reduce PFAS exposure and highlight opportunities to assess the effectiveness of workplace exposure reduction efforts. Our findings also suggest that screening tests for cognitive decline should be recommended by health care providers to firefighters, who are first responders.

## Supplementary information

Supplementary information

## Data Availability

The datasets generated during and/or analyzed during the current study are available from the corresponding author on reasonable request.
