# Supplementary material for: Association of per- and polyfluoroalkyl substances (PFAS) exposure with cognitive function in firefighters
Source: J Expo Sci Environ Epidemiol. 2026 Mar 25;36(4):656–66. doi: 10.1038/s41370-026-00861-y (PMC13331740; doi:10.1038/s41370-026-00861-y)
Supplement: Supplementary file 1 — Supplementary information [file 41370_2026_861_MOESM1_ESM.docx]

Table S1. Descriptive Statistics of 24 PFAS concentrations (pg/µL) in Firefighters of This Study and the NHANES Participants (2013-2014).

|  |  | **Firefighter Study** | | | | | | | | **NHANES** |
| --- | --- | --- | --- | --- | --- | --- | --- | --- | --- | --- |
|  |  |  | **DF %** | **LOD** | **Mean** | **SD** | **25^th^** | **50^th^** | **75^th^** | **Mean** |
| Perfluoro-n-carboxylic acids | Perfluoro-n-butanoic acid | PFBA | 7.81 | 0.05 | 0.33 | 0.08 | 0.27 | 0.33 | 0.37 | Not Measured |
|  | Perfluoro-n-pentanoic acid | PFPeA | 3.00 |  | 0.002 | 0.001 | 0.002 | 0.002 | 0.002 | Not Measured |
|  | Perfluoro-n-hexanoic acid | PFHxA | 19.75 | 0.05 | 0.05 | 0.06 | 0.01 | 0.03 | 0.05 | <LOD |
|  | Perfluoro-n-heptanoic acid | PFHpA | 100 | 0.49 | 0.40 | 0.31 | 0.19 | 0.31 | 0.50 | <LOD |
|  | Perfluoro-n-octanoic acid (Linear and Branched) | PFOA | 100 | 0.04 | 0.56 | 0.27 | 0.36 | 0.49 | 0.78 | 1.42 |
|  | Perfluoro-n-nonanoic acid | PFNA | 93.75 | 0.03 | 0.16 | 1.00 | 0.09 | 0.15 | 0.22 | 0.41 |
|  | Perfluoro-n-decanoic acid | PFDA | 77.78 | 0.08 | 0.07 | 0.05 | 0.03 | 0.08 | 0.10 | 0.19 |
|  | Perfluoro-n-undecanoic acid | PFUnDA | 26.56 | 0.04 | 0.08 | 0.06 | 0.03 | 0.08 | 0.10 | 0.13 |
|  | Perfluoro-n-dodecanoic acid | PFDoDA | 0 |  | <LOD |  |  |  |  | <LOD |
|  | Perfluoro-n-tridecanoic acid | PFTrDA | 0 |  | <LOD |  |  |  |  | Not Measured |
|  | Perfluoro-n-tetradecanoic acid | PFTeDA | 0 |  | <LOD |  |  |  |  | Not Measured |
| Perfluoro-alkyl-sulfonates | Potassium perfluoro-1-butanesulfonate | PFBS | 15.62 | 0.01 | 0.03 | 0.03 | 0.01 | 0.03 | 0.05 | <LOD |
|  | Sodium perfluoro-1-pentanesulfonate | PFPeS | 26.98 | 0.004 | 0.01 | 0.01 | 0.004 | 0.02 | 0.02 | Not Measured |
|  | Potassium perfluoro-1-hexanesulfonate | PFHxS | 100 | 0.01 | 0.90 | 0.57 | 0.49 | 0.76 | 1.19 | 1.08 |
|  | Sodium perfluoro-1-heptanesulfonate | PFHpS | 32.81 | 0.01 | 0.06 | 0.07 | 0.01 | 0.06 | 0.08 | 0.22 |
|  | Potassium perfluoro-1-octanesulfonate (Linear and Branched) | PFOS | 100 | 0.06 | 2.40 | 1.24 | 1.53 | 2.13 | 2.95 | 4.25 |
|  | Sodium perfluoro-1-nonanesulfonate | PFNS | 0 |  | <LOD |  |  |  |  | Not Measured |
|  | Sodium perfluoro-1-decanesulfonate | PFDS | 0 |  | <LOD |  |  |  |  | Not Measured |
| Polyfluoro (H-substituted)-alkyl-sulfonates | Sodium 1H,1H,2H,2H-perfluoro-1-hexanesulfonate | 4:2 FTS | 0 |  | <LOD |  |  |  |  | Not Measured |
|  | Sodium 1H,1H,2H,2H-perfluoro-1-octanesulfonate | 6:2 FTS | 0 |  | <LOD |  |  |  |  | Not Measured |
|  | Sodium 1H,1H,2H,2H-perfluoro-1-decanesulfonate | 8:2 FTS | 3.00 |  | 0.26 | 0.33 | 0.14 | 0.26 | 0.37 | Not Measured |
| Perfluoro-alkyl-sulfonamides | Perfluorooctane sulfonamide | FOSA | 15.00 | 0.03 | 0.29 | 0.22 | 0.12 | 0.24 | 0.44 | <LOD |
| N-substituted perfluoro-1-octanesulfonamidoacetic acid | N-methylperfluoro-1-octanesulfonamidoacetic acid (Linear and Branched) | MeFOSAA | 0 |  | <LOD |  |  |  |  | 0.13 |
|  | N-ethylperfluoro-1-octanesulfonamidoacetic acid (Linear and Branched) | EtFOSAA | 0 |  | <LOD |  |  |  |  | <LOD |

**Note**. DF: detection frequency. LOD: limit of detection. SD: standard deviation. NHANES: National Health and Nutrition Survey.

Table S2. Six PFAS Concentrations (pg/ µL) by Usage Frequency of Firefighting Foams (N=65)

| **How often did you use firefighting foam for a fire event last year?** | **N** | **Variable** | **Mean** | **SD** | **Range** |
| --- | --- | --- | --- | --- | --- |
| **Never** | 6 | \| PFOS \| \| --- \| \| PFOA \| \| PFHpA \| \| PFNA \| \| PFDA \| \| PFHxS \| | \| 2.53 \| \| --- \| \| 0.56 \| \| 0.43 \| \| 0.16 \| \| 0.07 \| \| 0.66 \| | \| 1.64 \| \| --- \| \| 0.40 \| \| 0.46 \| \| 0.18 \| \| 0.07 \| \| 0.33 \| | \| 0.66-5.35 \| \| --- \| \| 0.30-1.36 \| \| 0.10-1.30 \| \| 0.03-0.53 \| \| 0-0.16 \| \| 0.17-1.08 \| |
| **Sometimes/occasionally** | 32 | \| PFOS \| \| --- \| \| PFOA \| \| PFHpA \| \| PFNA \| \| PFDA \| \| PFHxS \| | \| 2.35 \| \| --- \| \| 0.55 \| \| 0.38 \| \| 0.13 \| \| 0.05 \| \| 0.94 \| | \| 1.09 \| \| --- \| \| 0.27 \| \| 0.25 \| \| 0.08 \| \| 0.05 \| \| 0.56 \| | \| 0.12-5.14 \| \| --- \| \| 0.15-1.24 \| \| 0.11-1.29 \| \| 0-0.25 \| \| 0-0.22 \| \| 0.18-2.75 \| |
| **Most of the time/frequently** | 22 | \| PFOS \| \| --- \| \| PFOA \| \| PFHpA \| \| PFNA \| \| PFDA \| \| PFHxS \| | \| 2.30 \| \| --- \| \| 0.57 \| \| 0.32 \| \| 0.16 \| \| 0.06 \| \| 0.74 \| | \| 1.28 \| \| --- \| \| 0.27 \| \| 0.22 \| \| 0.10 \| \| 0.05 \| \| 0.46 \| | \| 1.15-6.21 \| \| --- \| \| 0.24-1.13 \| \| 0.11-0.87 \| \| 0.01-0.41 \| \| 0-0.16 \| \| 0.21-1.70 \| |
| **Always** | 5 | \| PFOS \| \| --- \| \| PFOA \| \| PFHpA \| \| PFNA \| \| PFDA \| \| PFHxS \| | \| 4.03 \| \| --- \| \| 0.73 \| \| 0.56 \| \| 0.25 \| \| 0.07 \| \| 1.75 \| | \| 1.16 \| \| --- \| \| 0.25 \| \| 0.68 \| \| 0.08 \| \| 0.05 \| \| 0.67 \| | \| 2.44-5.02 \| \| --- \| \| 0.47-1.05 \| \| 0.17-1.58 \| \| 0.15-0.33 \| \| 0.02-0.13 \| \| 1.06-2.61 \| |

Note. SD: standard deviation. PFOS: potassium perfluoro-1-octanesulfonate; PFOA: perfluoro-n-octanoic acid; PFHpA: perfluoro-n-heptanoic acid; PFNA: perfluoro-n-nonanoic acid; PFDA: perfluoro-n-decanoic acid; PFHxS: potassium perfluoro-1-hexanesulfonate.

Table S3. Correlations between PFAS Analytes and Covariates

| PFAS Analytes | Age (years) | Race | Education | Annual Individual income | Frequency of seafood consumption in the past 30 days | Depressive symptoms | Smoking history | Number of weekly alcohol consumption | Years of service at the city level | Use of firefighting foam in the past year | Shower frequency after a fire event | Use of SCBA (yes/no) |
| --- | --- | --- | --- | --- | --- | --- | --- | --- | --- | --- | --- | --- |
| PFOA | 0.19 | 0.16 | 0.18 | 0.19 | 0.10 | -0.01 | 0.05 | 0.12 | 0.20 | 0.11 | 0.07 | -0.08 |
| PFOS | 0.19 | -0.05 | 0.21 | 0.14 | 0.38** | -0.05 | 0.05 | 0.14 | 0.22 | 0.15 | 0.20 | -0.17 |
| PFHpA | 0.02 | 0.06 | 0.10 | 0.10 | 0.16 | -0.13 | -0.07 | -0.19 | -0.02 | -0.01 | -0.05 | -0.13 |
| PFNA | 0.18 | 0.13 | 0.24 | 0.18 | 0.18 | -0.09 | -0.13 | 0.10 | 0.08 | 0.18 | 0.17 | -0.22 |
| PFDA | -0.08 | 0.05 | -0.32 | 0.21 | -0.15 | 0.16 | 0.16 | -0.15 | 0.03 | -0.10 | -0.01 | 0.03 |
| PFHxS | 0.23 | 0.04 | 0.01 | 0.20 | 0.23 | -0.08 | 0.02 | 0.29 | 0.17 | 0.20 | 0.19 | 0.06 |
| 24-PFAS Mixture | -0.03 | -0.10 | -0.07 | 0.03 | -0.06 | 0.07 | 0.17 | 0.07 | -0.03 | 0.10 | 0.15 | 0.18 |

Note. Race: Non-Hispanic White vs. others. Education: some college or less vs. Bachelor’s or higher. Annual individual income: ≤$60,000 vs. >$60,000. Depressive symptoms measured by CES-D: Center for Epidemiologic Studies Depression Scale, 10^th^ Edition. CES-D scores ≥16 or use of antidepressant medication indicated having depressive symptoms. Use of firefighting foam in the past year: never, sometimes/occasionally, most of the time/frequently, always. Shower frequency after a fire event: once vs. twice or more. PFOS: Perfluorooctanesulfonic acid (or perfluorooctane sulfonic acid). PFOA: Perfluorooctanoic acid. PFNA: Perfluorononanoic acid. PFDA: Perfluorodecanoic acid. PFHpA: Perfluoroheptanoic acid. PFHxS: Perfluorohexanesulfonic acid (or perfluorohexane sulfonic acid).


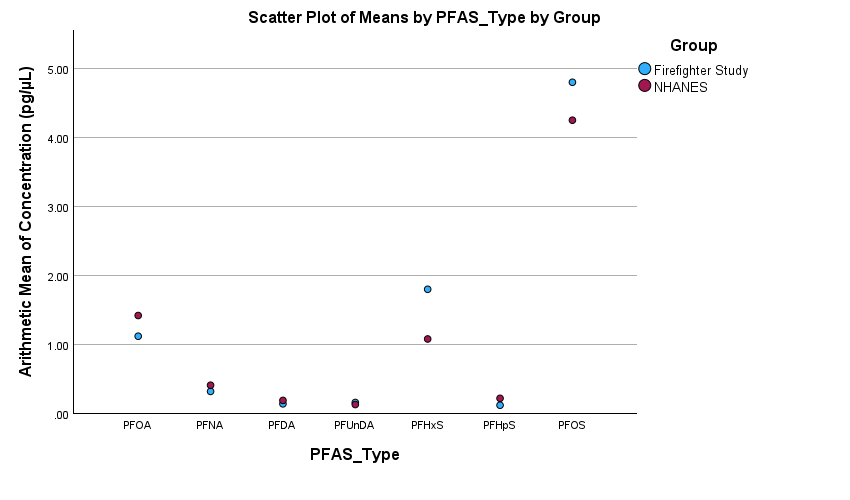


Figure S1. Average Concentration of 7 PFAS Analytes in This Firefighter Study and the National Health and Nutrition Survey (NHANES, 2013-2014). Conversion Factor (2:1 ratio serum/whole blood) Was Applied.

Global Cognition


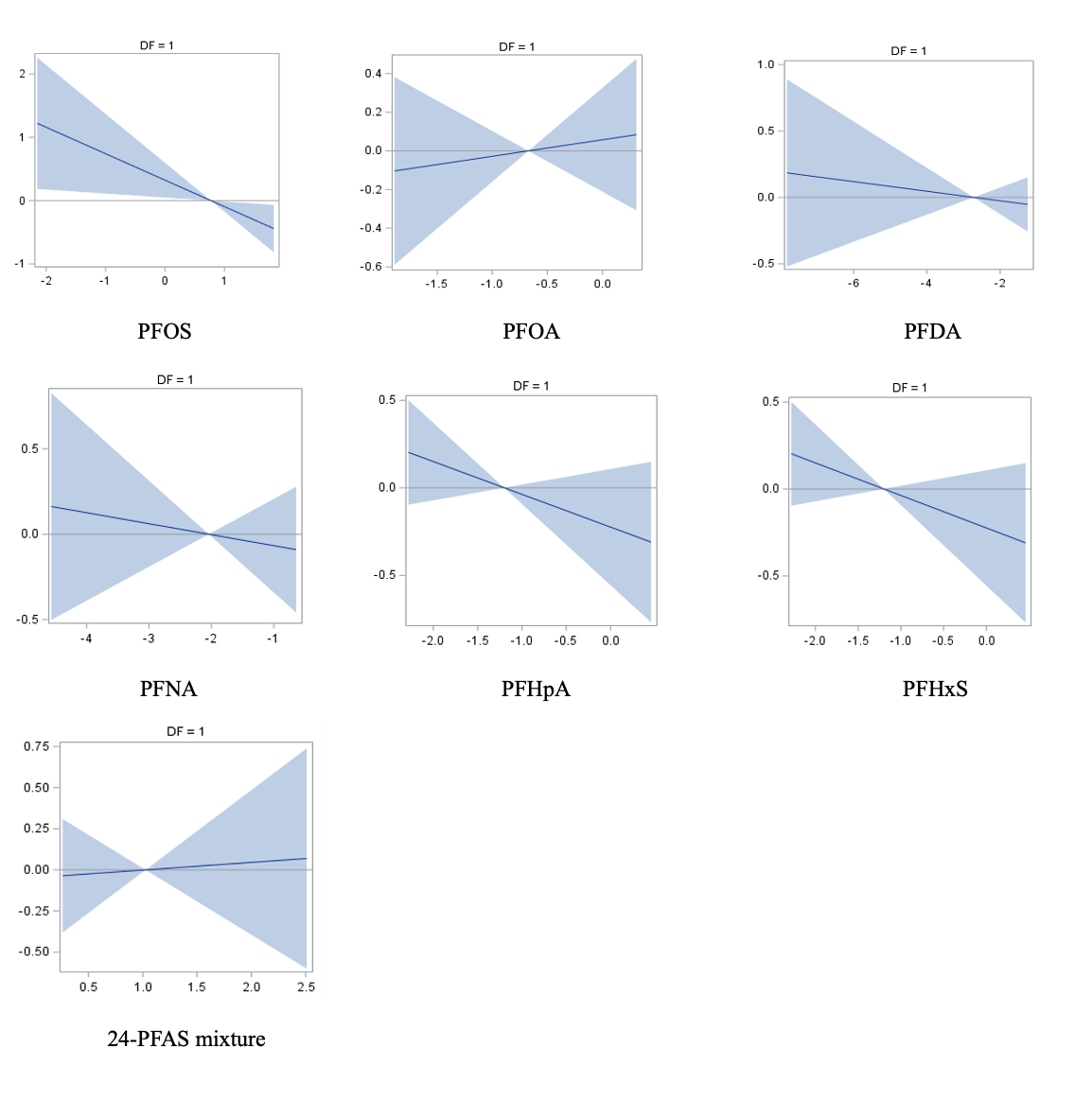


Processing Speed


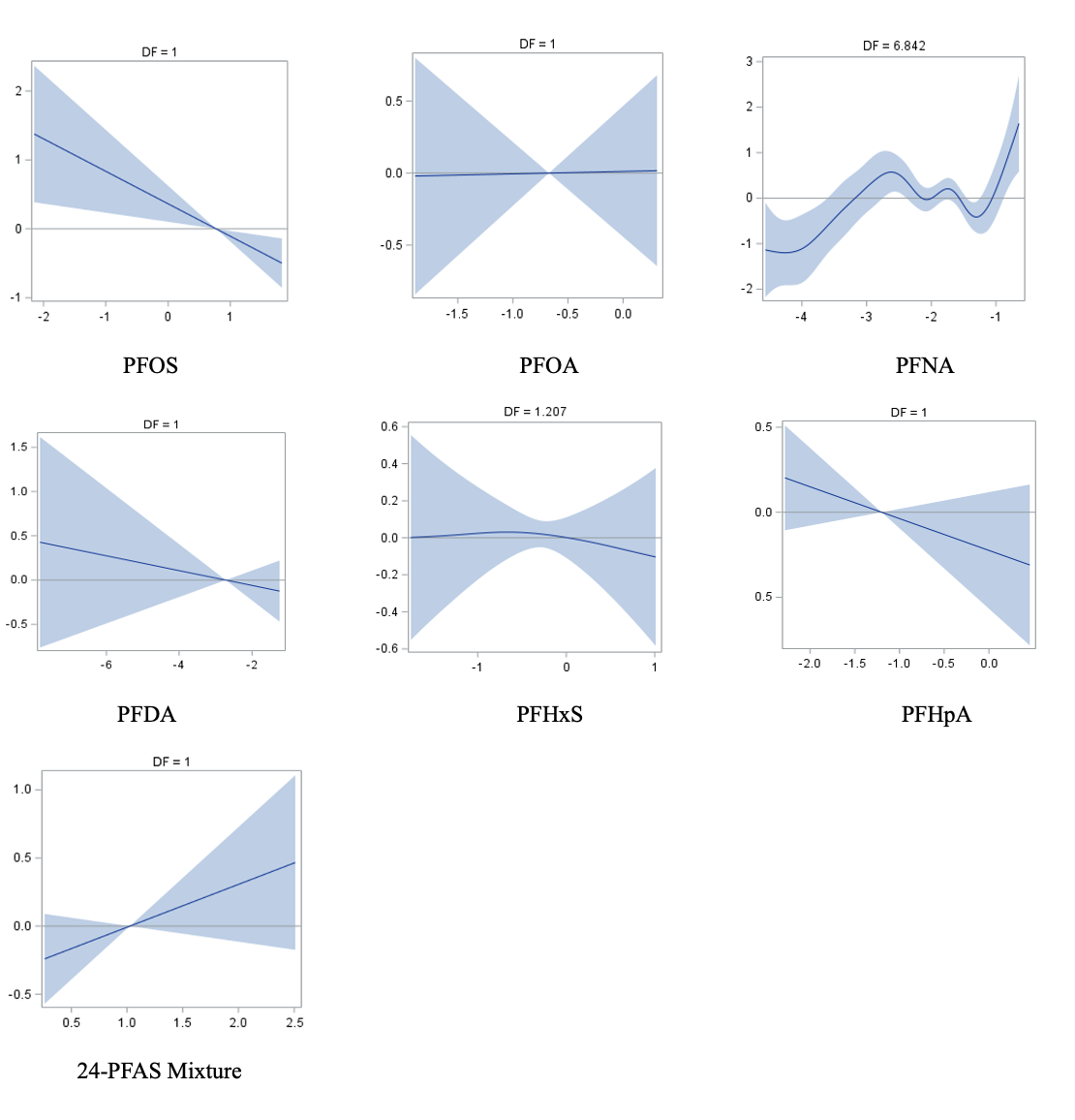


Figure S2. Smoothing plots of the associations of individual PFAS analyte and 24-PFAS mixture (transformed with natural logarithm) with global cognition and processing speed (composite z-scores). All models were adjusted for age, race/ethnicity, education, income, cigarette smoking history, alcohol consumption, seafood consumption, depressive symptoms, and occupational characteristics. Concentrations of individual PFAS analyte and the 24-PFAS mixture were fit using penalized splines in generalized additive models.


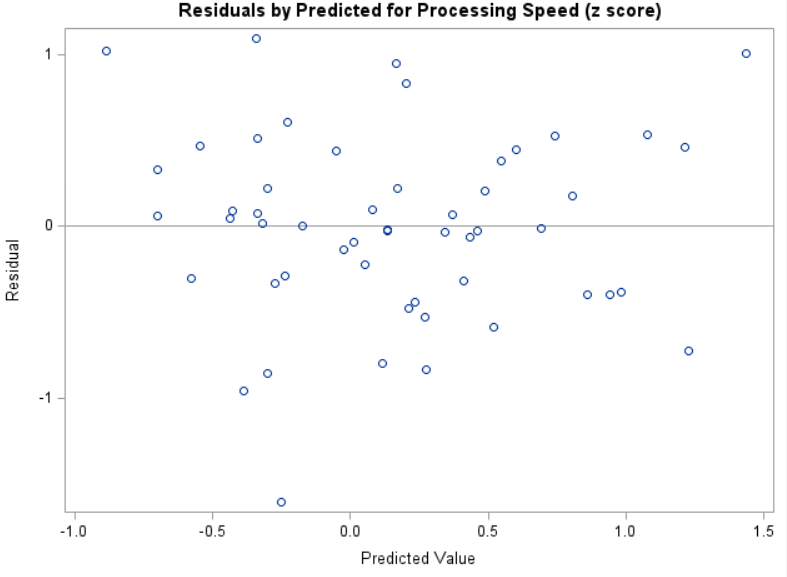


Figure S3. Residuals-versus-fitted-values diagnostic plot for linearity assumption between each PFAS analyte and processing speed.


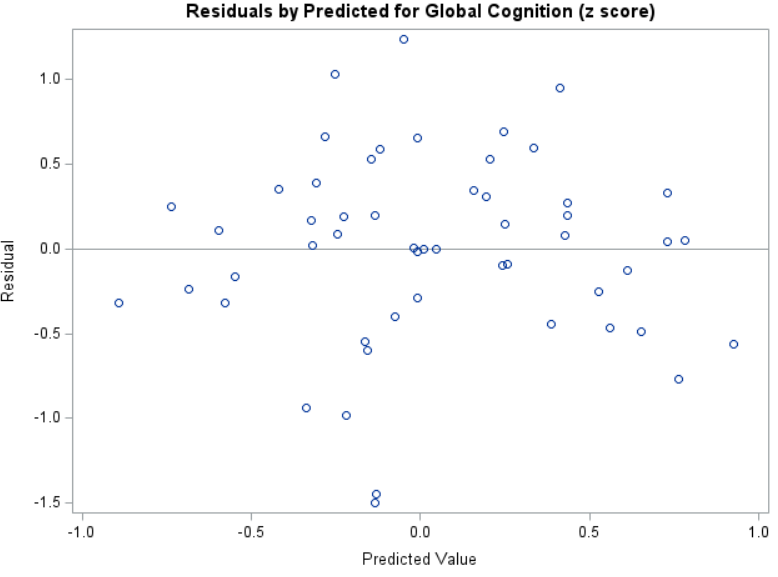


Figure S4. Residuals-versus-fitted-values diagnostic plot for linearity assumption between each PFAS analyte and global cognition.
